# Continuous Stroke Volume Estimation from Aortic Pressure Using Zero Dimensional Cardiovascular Model: Proof of Concept Study from Porcine Experiments

**DOI:** 10.1371/journal.pone.0102476

**Published:** 2014-07-17

**Authors:** Shun Kamoi, Christopher Pretty, Paul Docherty, Dougie Squire, James Revie, Yeong Shiong Chiew, Thomas Desaive, Geoffrey M. Shaw, J. Geoffrey Chase

**Affiliations:** 1 Department of Mechanical Engineering, University of Canterbury, Christchurch, New Zealand; 2 GIGA Cardiovascular Science, University of Liege, Liege, Belgium; 3 Intensive Care Unit, Christchurch Hospital, Christchurch, New Zealand; Medical University of Graz, Austria

## Abstract

**Introduction:**

Accurate, continuous, left ventricular stroke volume (SV) measurements can convey large amounts of information about patient hemodynamic status and response to therapy. However, direct measurements are highly invasive in clinical practice, and current procedures for estimating SV require specialized devices and significant approximation.

**Method:**

This study investigates the accuracy of a three element Windkessel model combined with an aortic pressure waveform to estimate SV. Aortic pressure is separated into two components capturing; **1**) resistance and compliance, **2**) characteristic impedance. This separation provides model-element relationships enabling SV to be estimated while requiring only one of the three element values to be known or estimated. Beat-to-beat SV estimation was performed using population-representative optimal values for each model element. This method was validated using measured SV data from porcine experiments (N = 3 female Pietrain pigs, 29–37 kg) in which both ventricular volume and aortic pressure waveforms were measured simultaneously.

**Results:**

The median difference between measured SV from left ventricle (LV) output and estimated SV was 0.6 ml with a 90% range (5^th^–95^th^ percentile) −12.4 ml–14.3 ml. During periods when changes in SV were induced, cross correlations in between estimated and measured SV were above R = 0.65 for all cases.

**Conclusion:**

The method presented demonstrates that the magnitude and trends of SV can be accurately estimated from pressure waveforms alone, without the need for identification of complex physiological metrics where strength of correlations may vary significantly from patient to patient.

## Introduction

Inadequate ability to diagnose cardiac dysfunction is prevalent in critical care [1], [2] and is a significant cause of increased length of hospital stay, cost, and mortality [3], [4]. However, detection, diagnosis and treatment of cardiac dysfunction are very difficult, with clinicians confronted by large amounts of often contradictory numerical data. Thus, it is important to synthesise raw clinical data such as blood pressure and heart-rate into useful physiological parameters such as stroke volume and contractility that can be used to improve diagnosis and treatment [5].

This goal can be accomplished using computational models and patient-specific parameter identification methods to unmask hidden dynamics and interactions in measured clinical data [6], [7]. This approach can create a clearer physiological picture from the available data and its time-course, making diagnosis simpler and more accurate, thus enabling personalised care [8], [9]. This approach also enables real-time, patient-specific monitoring which could allow faster diagnosis and detection of dysfunction [10].

Ventricular stroke volume (SV) measurements are essential for evaluating cardiovascular system (CVS) function [11]–[13]. Currently, SV can be estimated using non-invasive procedures including ultrasound, through moderately invasive methods including indicator dilution [14], to highly invasive direct measurement with admittance catheters. Only the latter can directly and continuously measure SV. Other modalities, including MRI and echocardiography, also do not necessarily show strong correlation to “gold standard” thermodilution [15]. Most methods require specialised equipment and/or personnel, and often only provide intermittent values of SV or measures of average SV over 10–15 seconds (e.g. cardiac output via indicator dilution) [16]. It is important to note that CO estimated by indicator dilution cannot capture transient effects or variability of SV on a beat-to-beat basis as these effects are averaged over 10–30 beats while the indicator transits the pulmonary circulation. Thus, SV is a much better metric for monitoring highly dynamic states, such as in shock, or when clinical interventions are made.

This paper presents a method for continuously estimating SV from aortic pressure measurements. Aortic pressure measurements are clinically available in the ICU and its continuous waveform signals allow SV to be determined on a beat-to-beat basis. Clinically, current discrete estimates of CO provide patient status at a particular point in time. However, the changes and trends of SV due to physiological changes and/or clinical interventions such as inotrope [17] or fluid therapy [18], are more important in optimizing clinical treatment. Thus, this method of identifying SV continuously could provide early identification of deteriorating patients, and could improve treatment and outcomes by providing a direct SV response to treatment [19].

This method utilizes the three-element Windkessel model to estimate stroke volume. The model used for this analysis is analogous to a Westkessel model where characteristic impedance (R) is added in series to a traditional Frank’s Windkessel model (parallel R-C circuit) [20]. The input to this RCR model is aortic blood pressure, and analysis of the pressure waveform with the model allows stroke volume estimation to be made. The unique aspect of this study is that the model only requires estimation of one of the three-element values for the estimation of SV and the other two parameters can be identified by the defined relationship within the model.

Thus, model complexity is optimised so that only a single parameter from the RCR model must be fixed for the estimation of SV, and the other two parameters are identified using measured data combined with the defined relationship within the model. This study investigates the accuracy of continuous beat-to-beat SV estimated using this method, and the sensitivity to the choice of fixed parameter. The model is validated against porcine experimental data where both ventricular volume and aortic pressure waveforms were measured simultaneously.

## Methodology

### Aortic Pressure Model

**Figure 1:**
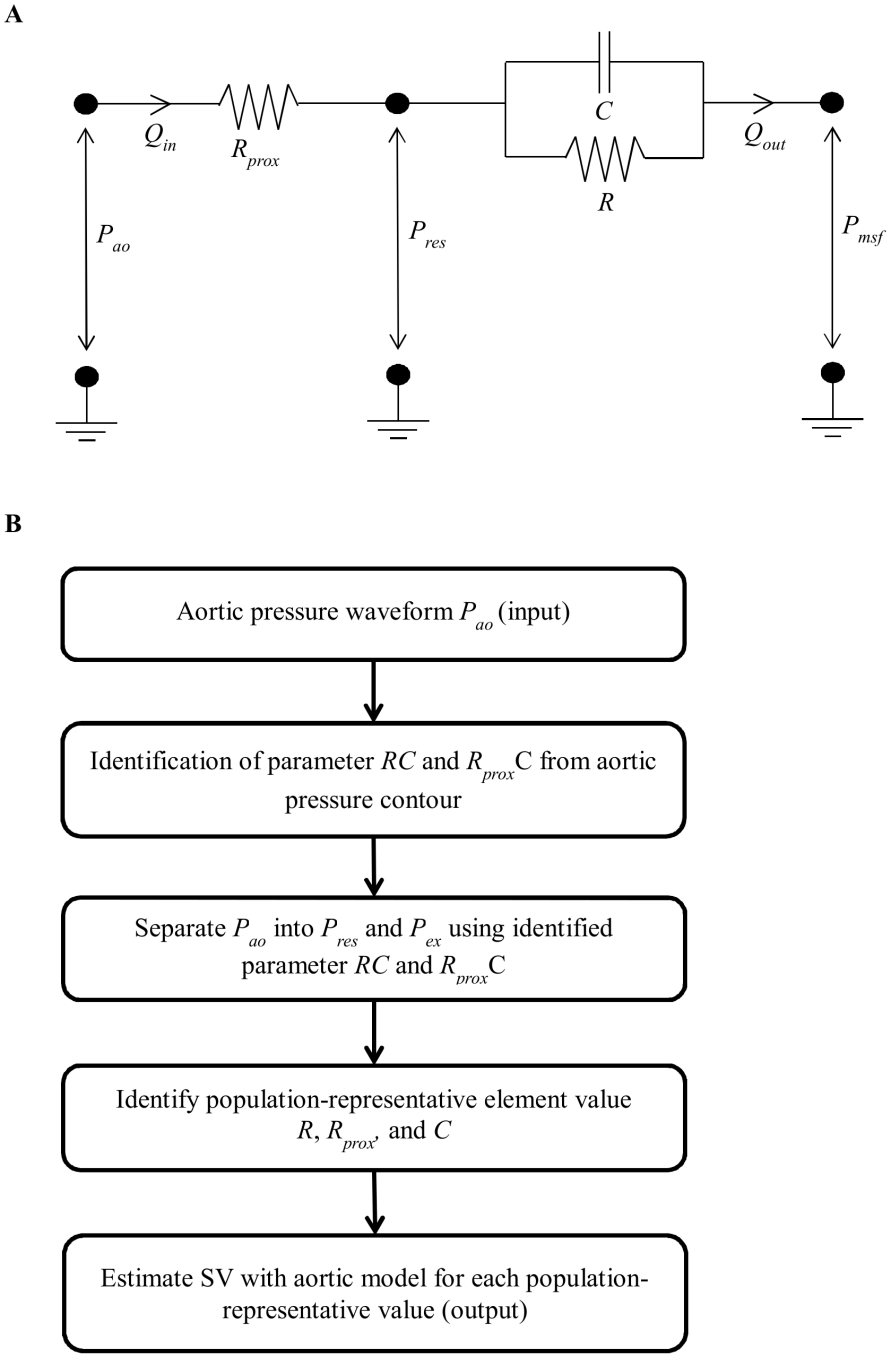
Aortic pressure model. A) Electrical analogy of the aortic model used in this study (systole). B) Flow chart showing stroke volume estimation process.

This study combines arterial Windkessel behaviour [21] and pressure contour analysis [22] to estimate left-ventricular stroke volume. This approach provides better representation of the arterial physiology and isolates aortic contour shapes to their corresponding arterial mechanical properties. Figure 1 presents electrical analogy of the model and schematic of the process used in this study.

The aortic pressure model used in this study is based on that of Wang *et al* [23]. This model proposes that aortic pressure *P_ao_* can be separated into two components, reservoir pressure, *P_res_*, and excess pressure, *P_ex_*. Reservoir pressure accounts for the energy stored/released by the elastic walls of the arterial system. Excess pressure is defined as the difference between the measured aortic pressure and the reservoir pressure that varies with time, *t*, and excess pressure can also be determined from ohm’s law as:
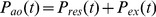
(1)




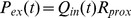
(2)Where *Q_in_* is flow entering aortic compartment from the left ventricle and *R_prox_* is the characteristic impedance relating inflow and excess pressure. In addition to the pressure relationships defined in Equations (1) and (2), the three element Windkessel theory was also applied [24], relating reservoir pressure and flow:



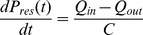
(3)

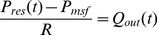
(4)


*C*, *R*, and *P_msf_* are defined as compliance, resistance and mean systemic filling pressure, respectively, and *Q_out_* is flow leaving the aortic compartment. In this case, aortic model parameters *C*, *R_,_* and *R_prox_* are assumed to be constant during single heartbeat.

By combining Equations (1)–(4), reservoir pressure can be expressed in terms of *P_ao_*_,_ *P_msf_*_,_ *R_prox_*_,_ *R*, and *C*:
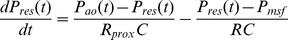
(5)


The analytical solution to Equation (5) for *P_res_* is defined:
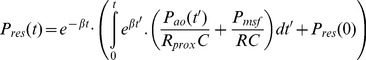
(6)Where *β = 1/R_prox_C+1/RC*. Equation (6) can be used to calculate reservoir pressure, which is dependent only on three parameters *RC*, *R_prox_C*, and *P_msf_*_._

The diastolic regions of the aortic pressure decay curve were used to identify exponential time decay constant *RC* and *P_msf_*. In this pressure region, inflow to the aortic compartment is assumed to be zero as a result of aortic valve closure and thus pressure decay results from only volumetric change of the arterial compartment [25]:
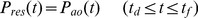
(7)Where *t_d_* and *t_f_* are the time of closure of aortic valve and total time for one cycle of heart beat, respectively. With this assumption, Equation (5) can be reduced such that *P_res_* is a function of only two parameters *RC* and *P_msf_*. Thus, the diastolic reservoir pressure can be expressed:



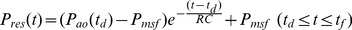
(8)Parameter value *RC* and *P_msf_* were identified by minimizing the discrepancy between measured diastolic pressure and estimated diastolic pressure from Equation (8). In this work, the start of diastole was defined by the time of the minimum rate of change of *P_ao_* [26].

**Figure 2:**
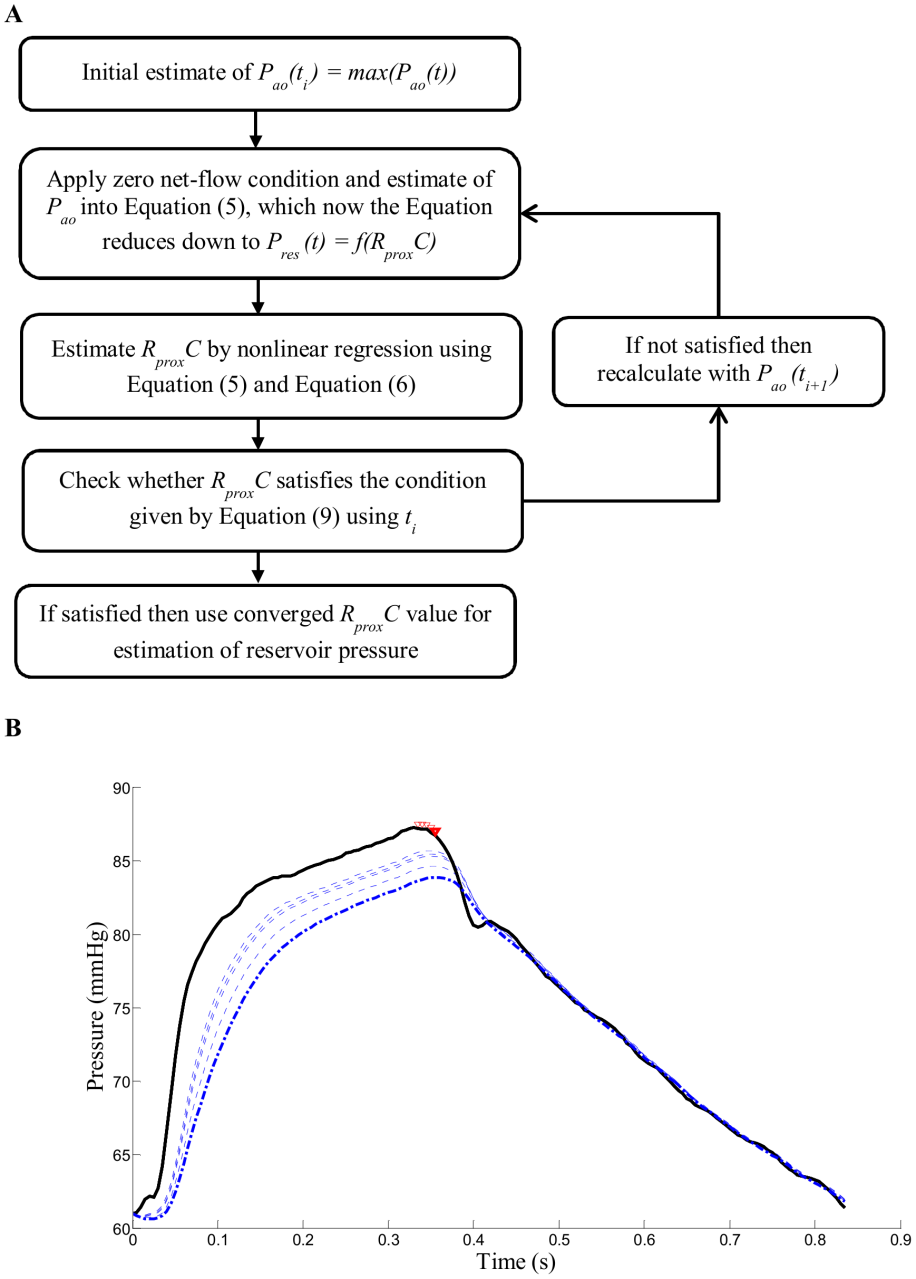
Reservoir pressure estimation process. A) flow chart showing iteration steps involved for identification of *R_prox_C*. B) graph showing measured *P_ao_* (thick solid black line), *P_ao_*(t_i_) value used at each iteration (red triangle points), and reservoir approximation through each iteration (thin blue dashed line) and final computed reservoir pressure using converged *R_prox_C* (thick blue dashed line).

For identification of *R_prox_C*, the systolic pressure waveform was used along with estimated values of *RC* and *P_msf_* from the previous steps. The identification of *R_prox_C* involves additional assumptions about the behaviour of the reservoir pressure curve. In particular, that zero net flow in the compartment occurs in the region between the point of maximum *P_ao_* and *t_d_*. This assumption comes from the knowledge that *P_ao_* increase is due to the increase in inflow and the compliant effects of the aorta. Therefore, there must exist a point where equilibrium of flow occurs when *P_ao_* is decreasing to assist valve closure. Using this additional information, and Equations (5) and (6), the value of *P_ao_* is iterated in this range to identify *R_prox_C* when the following condition is satisfied:
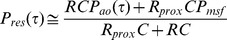
(9)Where τ is the time when inflow *Q_in_* is equal to the outflow *Q_out_*. Once the parameters *RC*, *R_prox_C*, and *P_msf_* are estimated, *P_ao_* is decoupled into reservoir and excess pressure using Equations (1) and (6). The process showing each identification step for *R_prox_C* and the converged reservoir pressure approximation using the *R_prox_C* value is illustrated in Figure 2.

**Figure 3:**
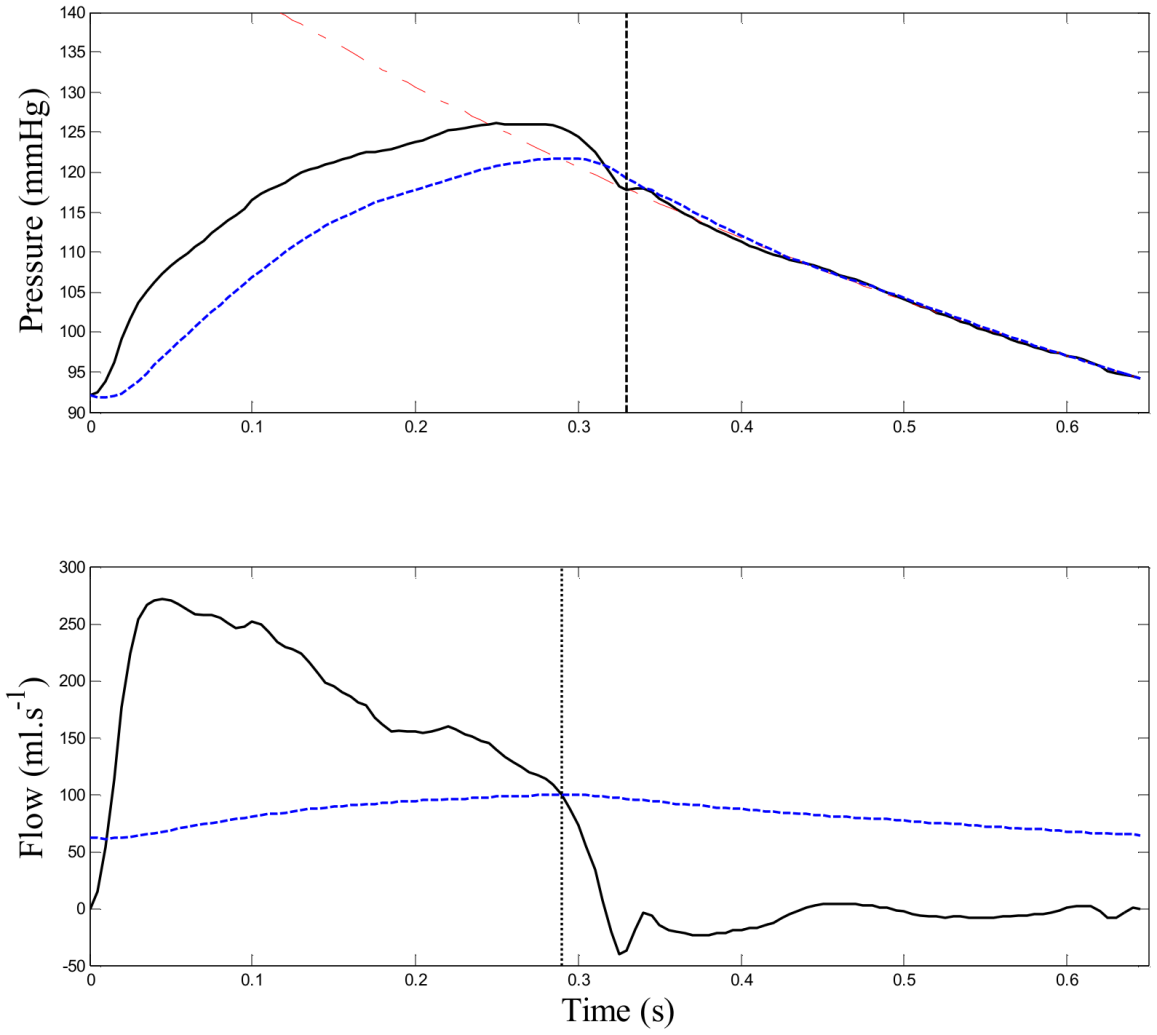
Example of computed reservoir pressure and aortic flow pattern. Top panel: example of aortic pressure separation showing estimated diastolic curve (red dash-dot line), reservoir pressure *P_res_* (blue dashed line), valve closure time *t_d_* (dashed black line), and measured aortic pressure *P_ao_* (solid black line). Bottom panel: Estimated aortic inflow *Q_in_* (solid black line), outflow *Q_out_* (dashed blue line), and zero net flow time τ (dotted black line).

Figure 3 shows example of measured aortic pressure and computed reservoir pressure using aortic pressure model. In addition, flow pattern computed using Equation (2) and (4) is shown.

### Porcine Trials and Measurements

#### Ethics Statement

All experimental procedure, protocols and the use of data in this study were reviewed and approved by the Ethics Committee of the University of Liege Medical Faculty (Approval number: 1230).

#### Experiments

This study used data from experiments performed on pigs at the centre Hospitalier Universitaire de Liege, Belgium. These experiments were primarily conducted to investigate respiratory failure, but extensive measurements of CVS variables were also recorded [27].

Experiments were performed on three healthy, female pure pietrain pigs weighing between 29–37 kg. During the experiments, each subject underwent several step-wise positive end expiratory pressure (PEEP) recruitment manoeuvres (RM). Increase in PEEP reduces systemic venous return to the right heart and as a consequence, left ventricular filling volume decreases causing reduction in SV [28]. Details of the experimental procedure are published elsewhere [27]. It should be noted that these experiment were performed with open chest. However, the chest of pigs 1 and 2 were held closed with forceps. Thus, the SV and arterial waveform were affected by direct pressure on the mediastinum area from expanding lungs.

Left and right ventricular volumes and pressures were measured using 7F admittance catheters (Transonic Scisense Inc., Ontario, Canada) inserted directly into the ventricles through the cardiac wall. Aortic pressure was measured with a 7F pressure catheter (Transonic Scisense Inc., Ontario, Canada) inserted into the aortic arch through the carotid artery. All data were sampled at 200 Hz and were subsequently analysed using Matlab (version 2013a, The Mathworks, Natick, Massachusetts, USA).

### Stroke Volume Estimation

The aortic pressure model cannot estimate SV unless one of the three Windkessel parameters (*R*, *C*, or *R_prox_*) is estimated. In this analysis, experimentally measured left-ventricular SV values enabled each optimal Windkessel parameter to be identified for all pigs by minimising the error between the measured and estimated SV. This approach can be thought of as a calibration of the parameters for converting relative changes identified from the aortic contour analysis into an absolute magnitude of SV. By fixing one of these parameters at its optimal value, and estimating stroke volume, the accuracy of SV estimation from the aortic pressure waveform can be evaluated. Applying this process for each of the Windkessel parameters enables evaluation of the SV estimation and the practicability of this method in each of the cases.

Identification of optimal, or ‘fixed,’ Windkessel parameters were conducted by grid-search within reported physiological ranges [29]. Values of resistance, compliance, and characteristic impedance, (*R_fixed_*, *C_fixed_*, *R_prox, fixed_*), were tested with resolution of 0.001 for each parameter:
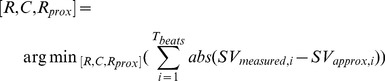
(10)Where *T_beats_* is total number of heart beats analysed from the experiment. Thus, the fixed values of these parameters represent the optimal values over all beats for all pigs. Stroke volume estimation was performed using identified fixed values of *R_fixed_*, *C_fixed_*, and *R_prox, fixed_* together with derived values of *P_ex_*, *P_res_*_,_ *RC*, and *P_msf_*.



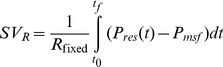
(11)

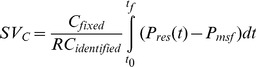
(12)




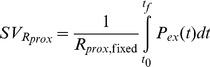
(13)Where *SV_R_*, *SV_C_*, and *SV_Rprox_*, represent the estimated SV using one of the pre-determined fixed values from Equation (10) for resistance, compliance, and characteristic impedance, respectively. Each of the SV values represents SV estimation with only the one parameter held constant within the three element Windkessel (*R*, *C*, or *R_prox_*) for the duration of the experiment.

### Data Analysis

**Figure 4:**
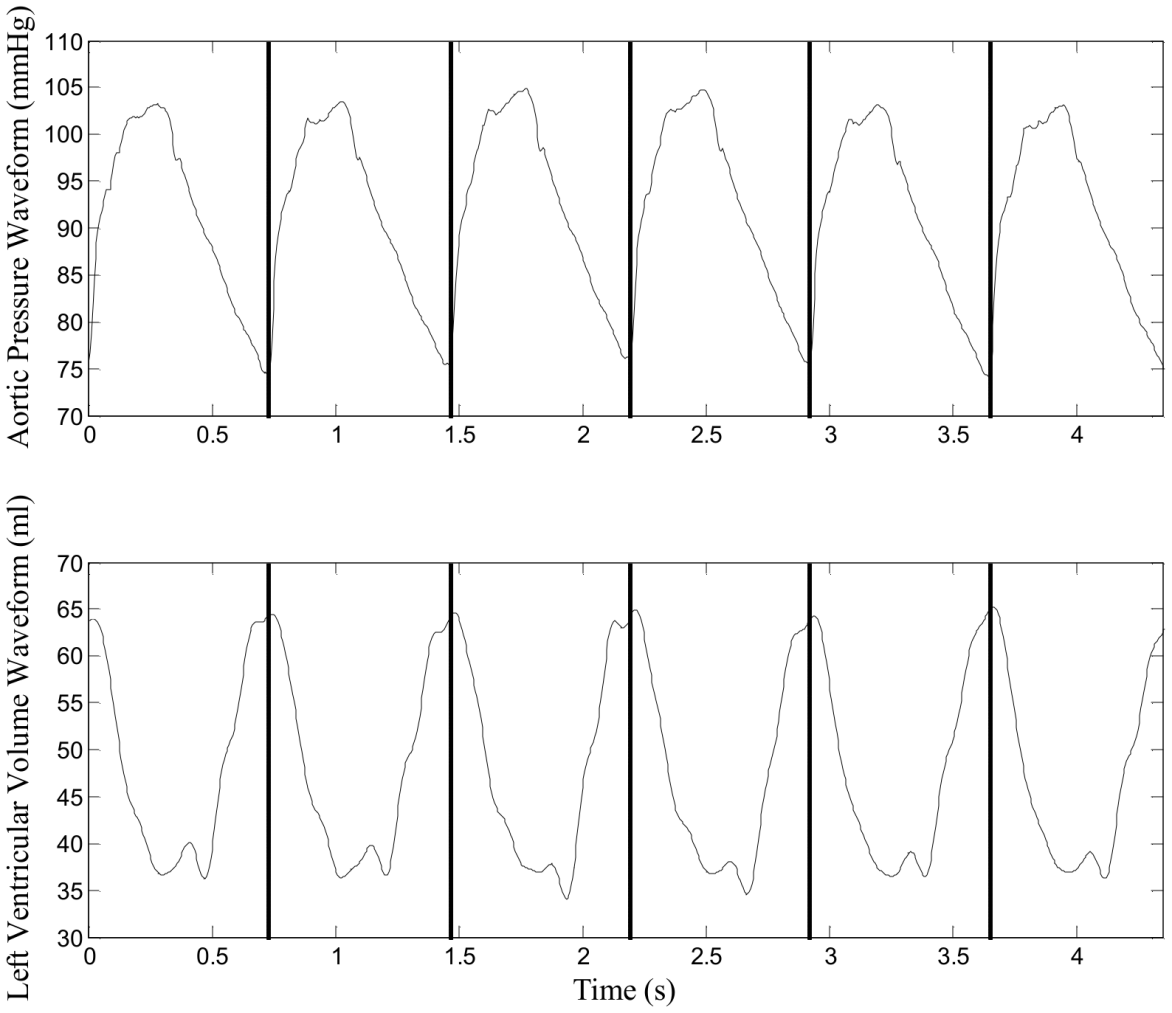
Example of measured aortic pressure and left ventricular volume waveform. Top Panel: example of measured aortic pressure waveform used for the model, black vertical line representing time at start of each heartbeat. Bottom Panel: example of measured Left Ventricular Volume Waveform used to determine SV for each heartbeat.

Measured aortic pressure and left ventricular volume waveform data were pre-processed by removing regions where obvious measurements error occurred due to equipment or catheter disturbance/failure. Using this pre-processed data, both aortic pressure and left ventricular volume waveforms were first split into individual heartbeat for the analysis. For each beat, SV were calculated as difference between maximum and minimum volume. Measured aortic pressure waveform was separated into reservoir and excess pressure components using the aortic model, prior to estimation of SV. Examples of measured aortic pressure and left ventricular waveforms are shown in Figure 4 and summary of analysed physiological range are presented in Table 1. In this study, agreement and distribution of differences between measured and estimated SV were shown with Bland-Altman plots and histograms. To assess trend accuracy, zero-lag cross-correlation coefficient values were calculated between measured and estimated SV in the recruitment manoeuver region where SV changes occur.

**Table 1:** Investigated range of physiological parameters, measured Mean Arterial Pressure (MAP), and SV.

Parameter	*MAP* (mmHg)	*SV* (ml)
**Physiological Range**	84.3 [59.6–112.5]	25.3 [13.6–39.2]

Data are presented as the median [5–95^th^ percentiles].

## Results

**Figure 5:**
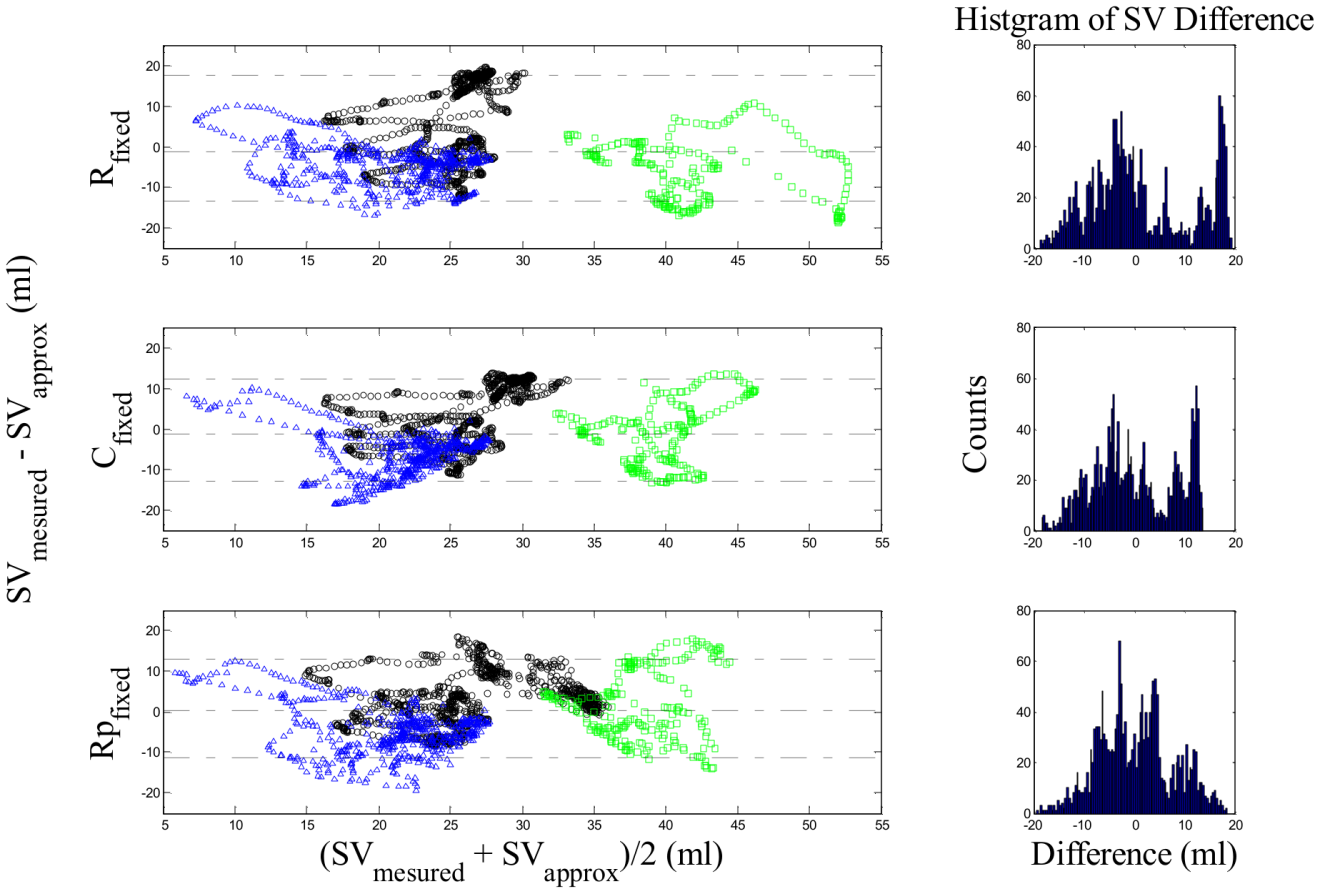
Bland-Altman plots comparing agreements between measured and estimated SV for all pigs using estimated population-representative values in Table 2 (Black circle: pig1, Blue triangle: pig2, Green square: pig3). Top Panel: Agreements of SV estimation using *R_fixed_* value in Equation (11). Middle Panel: Agreements of SV estimation using *C_fixed_* value in Equation (12). Bottom Panel: Agreements of SV estimation using *R_prox_, _fixed_* value in Equation (13).

**Figure 6:**
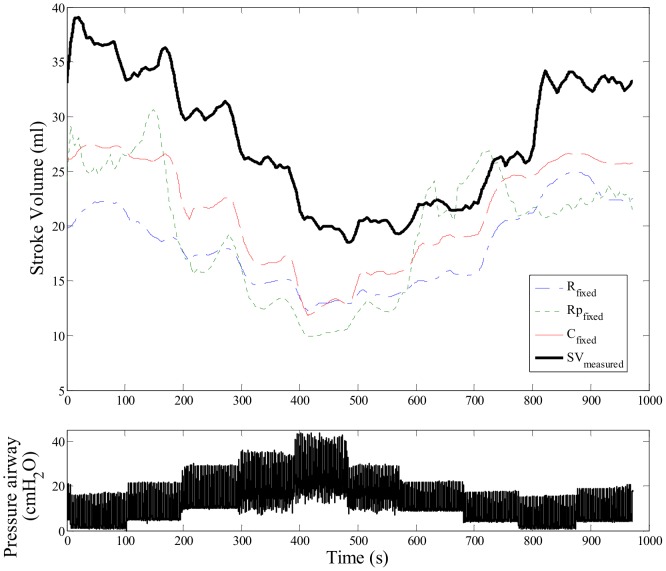
Example of measured SV and estimated SV during recruitment manoeuvers period. Top Panel: Example of SV estimation using different fixed parameters. Bottom Panel: simultaneously measured airway pressure to show the PEEP changes during recruitment manoeuvers.

The identified population-representative optimal parameter values, *R_fixed_*, *C_fixed_*_,_ and *R_prox, fixed_* for all pigs used in this study are presented in Table 2. Bland-Altman plots for all heart beats for each optimal parameter are presented in Figure 5, where there are approximately 300 to 1000 estimated SV values per pig. These plots compare directly measured values of SV to values estimated using Equations (11)–(13) with values from Table 2. Table 3 summarise the results of the Bland-Altman plots showing the accuracy of SV estimation using the model, and Table 4 summarise the calculated zero-lag cross-correlation coefficient values analysing the SV trend accuracy. In addition, example of the estimated SV compared to the measured SV in the recruitment manoeuver region is presented in Figure 6.

**Table 2:** Optimal parameter values in the three element Windkessel model for the estimation of SV.

Parameter	*R_fixed_* (mmHg.s/ml)	*C_fixed_* (ml/mmHg)	*R_prox, fixed_* (mmHg.s/ml)
**Optimized value**	1.6630	0.5330	0.0880

**Table 3:** Summary of Bland-Altman analysis from figure 4 for different fixed parameters.

Parameter	Bland-Altman results (ml)
R_fixed_(ΔSV)	−1.1[−13.3, 17.8]
C_fixed_(ΔSV)	−1.2[−12.8, 12.5]
R_prox, fixed_(ΔSV)	0.3[−11.2, 13.0]

Data are presented as the median [5–95^th^ percentiles].

**Table 4:** Summary of cross-correlation analysis for different fixed parameters in the recruitment manoeuvers regions.

Parameter	Zero lag cross-correlation coefficient
R_fixed_	0.67
C_fixed_	0.71
R_prox, fixed_	0.70

## Discussion

### Optimal Parameter Values

The optimal values for *R*, *C* and *R*_prox_ shown in table 2 were used to circumvent the structural model identifiability limitation of the Windkessel model and allow SV to be estimated from aortic pressure alone. By locating values that were representative of the population, the efficacy of the approach in application could be found. Patient specific parameters *R*, *R_prox_*, and *C* are identifiable if aortic blood velocity is obtained reducing the bias in the SV estimation, but at the expense of the need for additional device measuring such parameter.

Table 3 and Figure 5 demonstrate the ability to capture magnitude of SV. Across all beats and pigs, the median difference between measured and estimated SV was 0.6 ml, with a 90%-range of −12.4 to 14.3 ml. The median differences and 90%-ranges were similar for each fixed parameter. Thus, the proposed model is capable of estimating suitable SV by applying any of the three fixed parameters.

It should also be noted that cross correlation coefficients were above 0.65 for all cases. This result suggests that SV trends due to PEEP interaction were accurately captured, as shown in Table 4 and Figure 6. This outcome allows wider application. Specially, if any of the three parameters can be derived or estimated from additional, *a priori*, knowledge, a continuous SV estimation without external calibration (e.g. thermodilution) becomes possible. In addition, the presented SV estimation method could also be combined with other arterial mechanics models [30], [31] to further improve the estimation process.

### Stroke Volume Estimation

Pulse contour methods have been extensively studied as a means of estimating SV from continuous arterial blood pressure measurements [32]. However, conversions of pressure measurements to magnitude of flow are restricted to the assumptions made in the model-based approach. In particular, there are no direct relationships that can provide the scalars of volume from pressure measurements alone. As a consequence, the precision of SV estimations via model-based approaches are always limited to the assumptions made, such as strength of parameter correlations or stability of calibrated measurements [14], [33].

Historically, these models have evolved and increased in complexity to capture more accurate representations of realistic physiological phenomena [34]. More realistic models can provide better approximations of the SV and more detailed physiological insight. However, identification of the model parameters for these more complex models becomes much more difficult, if not impossible, eliminating their use in a practical application based patient-specific context, although this approach does increase their applicability as models for understanding [35], [36].

The aortic model presented in this study incorporates pressure contour analysis based on one dimensional flow in an elastic tube [37]. Despite the fact that the model is a zero-dimensional cardiovascular analysis, it can be treated as one segment from a whole network of arteries represented by multiple compartments, having many zero dimensional models connected together [38]. While the assumptions of this model are simplistic, they are made in consideration with what is available and practical clinically [39].

The model considers only the dominant influences that occur within a small compartment of aortic system. Thus, the model assumes arterial properties to behave in the same manner in between the aortic valve and where the measurements are taken. This assumption may produce error in the SV estimation, however, the numbers of influential physiological parameters that must be considered within this region are considerably fewer compared to the cardiovascular models comprising or considering the whole vascular systems. Therefore, the model is optimised for the purpose of estimating stroke volume from the pressure measurements.

This model-based approach also has an advantage that both arterial and heart properties could be analysed and hence, analysis of ventricular-arterial coupling is possible. Ventricular-arterial coupling is clinically important measure as knowing changes and trends of this parameter will provide patient response from inotrope and vasoactive drugs [40].

Combining the arterial Windkessel model and pressure contour analysis [41] increases the information that can be extracted from the aortic pressure contour [42]. This combination reduced the number assumptions required and allows non-fixed parameters to be constrained within the identified parameters *RC* and *R_prox_C*, helping to increase the ability of the model to match observed physiological conditions and thus estimate SV. In addition, beat-to-beat pressure contour variation due to altered arterial mechanics, *R*, *R_prox_,* and *C*, were related to correct corresponding pressure zones, enabling this model to more accurately capture SV variability from aortic pressure measurements alone.

The clinical applicability of the presented method currently relies on the availability of aortic pressure measurements. Arterial catheters are currently used in ICU patients who would benefit from continuous SV monitoring and aortic catheters are used in a number of these patients. However, arterial catheterization sites are determined by the perceived risk-to-benefit ratio and thus, increasing the benefit of obtaining aortic pressure waveform could reverse the trend turning potential risk into benefit [43], [44]. Additionally, models for estimating aortic pressure from radial or femoral artery pressure may be developed in the future which will enhance the clinical applicability of this approach.

### Limitations

The SV estimates were compared with directly measured left ventricular volumes, providing a true validation of the model accuracy to within errors using such measurements. Although left ventricular volume was measured directly and with the best available method, the sample size was small, with only 3 pigs being considered in this study. In addition, these measurements can be very sensitive to catheter location and condition in the left ventricle. However, this study analysed over 1500 heart beats, across a range of SV values induced by changes in PEEP. The range of SV analysed covers the expected normal range for most of the pigs [29]. Hence, there was sufficient data quality for the model to uniquely determine accurate parameter values. Thus, despite the small sample size, and other possible errors or variability, this study demonstrates the feasibility of accurately identifying SV using non-invasive, clinically available measurements.

In this experiment, changes to SV were induced by varying mechanical ventilation pressures, creating variation in the left ventricular preload. The effect of an increase or decrease in the thoracic cavity pressure alters the venous return to the ventricle and, as a consequence, stroke volume changes. In this study, the variations of systemic arterial mechanics are considered to be reasonably constant and the accuracy of the presented method may not be the same in cases where the subject’s hemodynamic conditions were significantly changed due severely diseased condition or extreme levels of care such as high ventilation pressures.

A final limitation of this aortic model is that the separation of aortic pressure waveform into reservoir and excess pressure represents a separation of forward and backward travelling waves. This assumption is based on the work of Wang *et al* [23], which shows proportionality in the inflow *Q_in_* and excess pressure *P_ex_*. This rationale may not hold for subjects with extraordinary or highly dysfunctional physiological conditions.

## Conclusion

Physiological models are simplified representations of reality that can provide clinicians with information for decision making, without the need for additional invasive direct measurement. The models presented in this study show the potential for continuous, accurate SV measurements using measurements typically available in the intensive care unit. This method of obtaining SV from aortic pressure waveform alone is more adequate for relating our knowledge about circulatory physiology to blood pressure values. The study showed SV variations across all beats and pigs, can be captured with precision of median difference between measured and estimated SV of 0.6 ml, with a 90%-range of −12.4 to 14.3 ml. Moreover, the agreement of SV trends showed cross correlation coefficient of above 0.65 for all cases. Thus, the aortic model is capable of estimating SV in both healthy and acute respiratory distress syndrome (ARDS) states with suitable accuracy, and with good trend accuracy in response to changes in treatment. Hence, this aortic model and approach shows the ability for extending our current understanding of the CVS mechanics, and to optimise real-time diagnosis and cardiovascular therapy.
